# 

**Published:** 1851-12

**Authors:** 


### INDEX TO VOL. VIII.

### [THIRD SERIES.]

A

Abstinence, remarkable case of...355
A case of retrocession of the cutaneous efflorescenee in rubeola,..........559
A case of rupture of the intestines,..........337
A case of small pox, third attack...360
American medical association, 85, 276, 366
American medical productions,....458
American scientific association,....88
Amputation, cases of..........429
Anderson's case of remarkable migrations of a pin and needle,...200
Anderson's remarks on nitrate of silver in Leucorrhea,........294
An important and dangerous surgical operation, from note book of P. K.,..........369
A new epidemic exanthem,........378
Austrian remedy for cholera,.....449

B

Bad midwifery,..........182
Bartlett, Professor..........89
Bee, the honey as a dieuretic.......123
Bell's case of poisoning, by sulphurretted hydrogen gas,....... 9
Bell's synopsis of practical medicine,..........378
Books received,.......... 91

C

Cannabis Indica, a substitute for Ergot,..........397
Case of poisoning by laudanum,...172
Case of gun shot wound,.........255
Case of popliteal aneurism cured by compression,..........257
Case of prolapsus vesica..........338
Case of puerperal convulsions,....340
Case of stricture of the bowels, ...343
Case of progressive and morbid diminution of the whole body,...347
Case of rape,..........452
Cases of amputation, ..........429
Charge against a medical practitioner,..........165
Chipley on medical journals and medical criticism,..........277
Chipley on vital statistics of Fayette county,..........461
Chloroform, purity and use of....153
Chloroform in Gonorrhea,.......250
Cholera in Louisville, Ky.,.......269
Cholera Infantum, quinine in treatment of..........359
Circular to the physicians of Kentucky and Tennessee,......183
Circular of the American medical association,..........276
Circular, of the committee of American medical association, on Hernia,..........539
Circulation—Hepatico-renal,.....247
Clinical observations in private practice,..........185
Cobb's translation of pharmacy of the Greeks and Romans,.....192
Cod-liver oil in scrofulous affections and consumption, ........250
Coilege of physicians of Philadelphia,.......... 90
Collodion for ingrowing nail,.....358
Combustion spontaneous, translated by P. K.,.......... 93
Complete absence of the uterus,..171
Consumptive patients, peculiar appearance of the gums,.......384
Convention, medical..........273
Convention of medical delegates, ..368
Cooking and preserving meat, ....262
Creasote in diarrhea, ..........360

Criticism, medical ..............277
D
Death of Dr. S. G. Morton,....... 90
Deficient lactation, remedy for ....382
Demonstrative midwifery,.........357
Diagnosis of fevers,..............354
Diarrhea, creasote for............360
Discoverer of etherization,.........458
Diseases malignant, surgical operations for .................. 92
Dislocation of the femur, new method of reducing ............391
Dysentery, treatment of....1, 123, 201
E
Efflorescence cutaneous, retrocession of, in rubeola, by Dr. Spillman,......................259
Effects of aneurism, ..............386
Elixer of opium, substitute for ....170
Epidemics of Kentucky and Tennessee, ................... 89
Epidemic exanthem, ..............378
Ergot, substitute for ..............397
Essays, prize......................362
Ether, discovery of ...............458
F
Fayette county, vital statistics of.......................453, 461
Fear sudden, hair blanched by....363
Fee's treatment of dysentery, &c...123
Femur dislocation of, new method of reducing,..................391
Fenner's southern medical reports,.........................367
Fevers, diagnosis of ..............354
Fever malignant scarlet, yeast in..882
Fever pnerperal, prophylaxis of...404
Fistula vesico-vaginal, operation for...........................398
Flint's Professor Austin, fever reports, .......................457
G
Gas snlphuretted hydrogen, poisoning,........................ 19
German views of the operation of turning,..................... 84
Gonorrhea, chloroform in .........250
Gross on the urinary organs,......181
Gums of consumptive patients, peculiar appearance of..........384
Gun shot wound in the spine, ....255
H
Hair blanched by sudden fear, ....363
Hardin on dysentery, ............. 1
Haskins' clinical observations,....185
Health of Louisville, 90, 181, 364, 456
Hepatico-renal circulation, ........247
Hernia, strangulated..............191
Hernia cure of, circular of the American medical association,...000
Honors of war,...................457
Hydrogen sulphuretted gas,....... 19
I
Important surgical operation, .....369
Incontinence of urine, ............172
Infantum, cholera, quinine in.....359
Influence of the poison of northern rattle-snakes on plants,.......356
Ingrowing nail, collodion for......358
Irritation, ovarian.................394
Institution, Smithsonian........... 87
Intestines, rupture of .............337
J
Journals, medical..................277
Journal, southern medical and surgical,.......................174
K
Kenney's remarks on dysentery,...201
Kentucky State medical society,...................182, 457
Kentucky and Tennessee, epidemics of...................... 89
Kentucky and Tennessee, physicians of, circular to ...........183
L
Lactation defective, remedy for....382
Laudanum, poisoning by .........172
Lectures, preliminary .............364
Lectures, medical.................456
Lenoir's surgical operation,........369
Leucorrhea, nitrate of silver in ....294
Lithotrity and Lithotomy, by T. S. Bell, M. D.,..................435
Louisville, cholera in ............269
Louisville University,.... 184, 276, 361
Louisville, health of..90, 181, 364, 45[illegible]
M
Malarial disease, appearance of tongue in.......................109
Malignant disease, results of surgical operations in............ 92
Malignant scarlet fever, yeast treatment, ......................382
Malignant pustule,................401
Mal-practice, suit for.............168
Man temperature of, within the tropics, .......................353
Measures for combatting sterility,..173
Meat, preservation of.............262
Medical association, American.... 61
Medical association, American circular of.......................276
Medical association, American....366
Medical convention, ..............273
Medical convention delegates,....368
Medical society, State.............262
Medical society of Tennessee,.....275
Medical State society of Kentucky,.457
Medical practitioner, charge against 165

Medical Journals and medical critics, ........................277
Medical lectures, .................456
Medical journal of jurisprudence,..455
Medical association, American—committee on the cure of reducible hernia,..................539
Medical American productions,.....458
Medicine, practical ..............378
Medicines, patent..................459
Midwifery, bad....................182
Midwifery, demonstrative .........357
Morbid diminution of the whole body, ..........................347
Morton, Dr. S. G., death of ....... 93
McMunn's elixer of opium, substitute for ......................170

N

Nail, ingrowing...................358
Nelson's Northern Lancet,........455
New method of reducing dislocations of the femur,...............391
Nitrate of silver in Leucorrhea,....294

O

Obituary,.........................368
Occlusion of the vagina,..........245
On the purity and use of chloroform, ..........................153
On a substitute for McMunn's elixer, .........................170
Operation for vesico-vaginal fistula, 398
Organs urinary, Gross on ........181
Osborn on the tongue in malarial disease,......................109
Ovarian irritation,................394
Owen on strangulated hernia, .....191

P

Patent medicines, ................459
Patients in consumption, appearance of the gums, .............384
Pertussis,.........................403
Pharmacy of the Greeks and Romans, ........................192
Physicians college of, Philadelphia, 90
Physiology of woman,.............247
Physicians of Kentucky and Tennessee, circular of .........183
P. K.'s translations from Archives Generales,...................... 93
P. K.'s notes of a surgical operation, ........................369
Pin and needle, migrations of .....200
Plant, effect of rattle-snakes on....356
Poisoning with sulphurretted hydrogen gas,...................19, 91
Practice private, clinical observations in ......................185
Practitioner of medicine, charge against, ........................165
Practical medicine, ................378
Preservation of meat,.............262
Prize essays,......................362
Proceedings of a convention of physicians in Frankfort, Ky.,....372
Prof. Bartlett, ................... 89
Prolapsus vesicæ,.................338
Prophylaxis in puerperal fever, ...404
Puerperal convulsions,............340
Puerperal fever, ..................404

Q

Quinine in cholera infantum,.359, 362
Quinine in urticaria,.............402

R

Rogers' Lewis M. D., lecture on sanitary reform, ................497
Registration law in Pennsylvania,.442
Rape, alleged ....................264
Rape, ............................452
Remarks on the tonge in malarial disease, ....................109
Remarks on dysentery, ...........201
Remarks on nit. Argenti, in Leucorrhea, ...................294
Remarkable migrations of a pin and needle, ....................200
Reports, Southern Medical........367
Results of surgical operations for malignant diseases, ............ 92
Retraction, the ...................182
Retrocession of cutaneous efflorescence in rubeola,..........259
Rupture of the intestines,.........337

REVIEWS.

Materia Medica and Therapeutics: with ample illustrations of Practice in all the departments of Medical Science, and very copious notices of Toxicology, suited to the wants of medical students and practitioners. By Thomas D. Mitchell, A. M., M. D., Professor of the Theory and Practice of Medicine, in the Philadelphia College of Medicine, formerly Professor of Chemistry and Pharmacy in the Medical College of Ohio; Professor of Chemistry and Materia Medica in the Medical Department of Transylvania University, Lexington, Ky.; Lecturer on Obstetrics and Diseases of Women and Children; Author of "Elements of Chemical Philosphy," "Hints to Students," &c., &c., &c.,.................... 37
On Diseases of Menstruation and Ovarian Inflammation in connexion with Sterility, Pelvic Tumors, and Affections of the Womb. By Edward John Tilt,

M. D., Physician to the Farrington General Dispensary, and to the Paddington Free Dispensary for the Diseases of Women and Children. 'Omne animal ab ovo.' 126
Operative Surgery. By Frederic
The Dissector, or, Practical Surgical Anatomy. By Erasmus Wilson, author of a "System of Human Anatomy." With one hundred and fifteen illustrations. Edited by Paul B. Goddard, M. D. A new and improved edition. 148
C. Skey, F. R. S., ............ 142
A Treatise on Dislocations and Fractures of the Joints. By Sir Astley Cooper, Bart., F. R. S., Surgeon to Guy's Hospital.—With additional observations, and a memoir of the author. A new American edition, ............ 148
On the Theory and Practice of Midwifery. By Fleetwood Churchill, M. D., M. R. I. A., etc. With notes and additions, by D. Francis Condie, M. D., Secretary of the College of Physicians, etc., etc. With one hundred and thirty-nine illustrations. A new American, from the last improved Dublin edition............ 150
Urinary Deposits; their Diagnosis, Pathology, and Therapeutical Indications. By Golding Bird, A. M., M. D, F. R. S., F. L. S., etc., etc. Second American, from the third revised and enlarged London edition............ 151
A Practical Treatise on Diseases and Injuries of the Urinary Bladder, the Prostate Gland, and the Urethra. By S. D. Gross, M. D., Professor of Surgery in the University of Louisville, Member of the American Medical Association, author of Elements of Pathological Anatomy, etc., etc., ............ 206
Description of fifteen new Species of Crinoidea from the Sub-carboniferous Limestone of Iowa, collected during the United States Geological Survey of Iowa, Wisconsin, and Minnesota, in the years 1848—'9. By David D. Owen. M. D., and B. F. Shumard, M. D. ............ 238
The Outlines of General Pathology. By M. L. Linton, M. D., Professor of the Theory and Practice of Medicine in the Medical Department of the St. Louis University, ............ 243
Lectures on the Eruptive Fevers, as now in the course of delivery at St. Thomas's Hospital, London. By George Gregory, M. D., Fellow of the Royal College of Physicians of London; Physician to the Small Pox and Vaccination Hospital at Highgate; Corresponding Member of the National Institute of Washington, etc.—First American edition, with numerous additions and amendments by the Author, comprising his latest views. With notes and an appendix, embodying the most recent opinions on exanthematic pathology; and also statistical tables, and colored plates. By H. D. Bulkley, M. D., Physician of the New York Hospital; Fellow of the New York College of Physicians and Surgeons, etc., etc. ............ 298
A Practical Treatise on the Diseases and Injuries of the Urinary Bladder, the Prostate Gland, and the Urethra. By S. D. Gross, M. D., Professor of Surgery in the University of Louisville, Member of the American Medical Association, author of Elements of Pathological Anatomy, etc., etc. ............ 314
Surgical Anatomy. By Joseph Maclise, Surgeon. With colored plates............ 409
Southern Medical Reports. Edited by E. D. Fenner, M. D., of New Orleans, ............ 418
Elements of General and Pathological Anatomy, presenting a view of the present state of knowledge in these branches of Science. By David Craigie, M. D., F. R. S. E. 421
Special Anatomy and Histology.—By Wm. E. Horner, M. D. .... 422
The Laws of Health, in relation to mind and body; a series of Letters from an old Practitioner to a Patient. Bo Lionel John Beale, M. R. C. S. ............ 423

S

Sanitary reform, ............ 178
Scarlet fever, ............ 382
Scientific association, American... 88
Silliman, Professor ............ 368
Silver, nitrate in Leucorrhea, .... 294
Small pox, case of............ 360

Small pox, treatment of...........383
Small pox and vaccination, .......448
Smithsonian Institute,............. 87
Smith, J. V. C., of Boston, .......364
Society Medical, of Tennessee,...275
Society Medical, of Kentucky, ....467
Southern Medical Reports,........367
Southern Medical and Surgical Journal, ......................174
Spontaneous combustion, ........ 93
Statistics, the vital of Fayette county, ..................453, 461
Statistics, the vital of Scott county, Ky., ...................... 7
Sterility, measures for combatting.173
Stricture of the bowels, ..........343
Substitute for McMunn's elixer of opium, ....................170
Substitute for ergot, .............397
Suit for mal-practibe, .............168
Sulphurretted hydrogen gas poisoning, ...................19, 91
Surgical operation at Neckar Hospital,....................369
Sutton, Dr. W. L. on the vital statistics of Scott county, Ky., 7, 490
T
Tartar, aqueous solution of.......451
Temperature of man within the tropics,........................353
The American medical association, 61
The retraction,...................182
The honors of war,...............457
The ether discovery, .............458
The case of sulphuretted hydrogen gas poisoning,............ 19
Tongue in malarial disease, ......109
Tracheotomy, .................... 91
Transactions of the college of physicians of Philadelphia, ..... 90
Treatment of small pox,...........383
Treatment of urticaria,............402
Treatment of dysentery,...1, 123, 101
Trial for alleged rape,.............264
Turning, German views of........ 84
U
University of Louisville,.184, 276, 361
University of Pennsylvania, ......362
Urea, its tests and origin,..........350
Urine, incontinence of ............172
Urinary organs, Gross on ........181
Urticaria, treatment of...........402
Use of chloroform, ...............153
Use of aqueous solution of tartar, .451
Uterus, absence of ...............171
V
Vaccinatiou and small pox, .......448
Vagina, occlusion of..............245
Vesicæ, prolapsus ...............338
Vesico-vaginal fistula,............394
Viability, early....................396
Vichy water, artificial ............173
Views of the Germans on the operation of turning, .......... 84
Vital statistics of Scott county, by W. L. Sutton, M. D.,.... 7, 490
Vital statistics of Fayette county,..453
Vital statistics of Fayette county, by W. S. Chipley, M. D.,......461
W
War, honors of ..................457
Water warm in colic,.............123
Water, Vichy artificial............173
Woman, physiology of............247
Wound of the spine,..............255
Y
Yandell's D. W. report of proceedings of the medical convention, ......................372
Yeast in malignant scarlet fever,...382

☞ We publish the following—not in anger, for we are used to all sorts of people—but to give our readers an instance of the manner in which we are sometimes treated. The terms of this Journal, it must be well known by all of its subscribers, are "cash in hand;" but we have been in the habit often of granting to our patrons the most lengthened indulgence. Occasionally, however, we look over our books and give notice to our subscribers of the state of their accounts, accompanied by a request to remit to us by mail at our risk. Accordingly we have lately had occasion, several times, to send out the following account:

Dr. J. M. Jones, Adairville, Ky.,

*To Office of the Louisville Journal, Dr.*

To subscription to the Western Journal of Medicine and Surgery
from January 1st, 1843 to January 1st, 1852,.............................$45 00
Credit by cash,......10 00

$35 00

In reply we have received the following:

"*Adairville, Ky., Nov. 19th*, 1851.

"Inclosed please find a pair of duns, I send them thinking you might become scarce of that material, and do not wish you to put yourself to the expense of having more printed. Whenever it *suits* my convenience to *remit* for the Journal I shall then be pleased to do so. Should this not accord with your notions you can let us know.

"I will just say to you I do not take your duns out of the office, there are some remaining in the office at this time—I have taken out these two, thinking you might want them, I will say to you, I shall take no more out of the office—when I can conveniently send the money for the Journal, I may do so. I am good for small contracts.

"Yours respectfully,

"J. M. JONES."

If there are any more Dr. Jones' upon our subscription list, we will take it as a favor, if they will let us know it.

### OUR DELINQUENT LIST.

IF we were to publish the names of our delinquent subscribers as faithfully as we publish the receipts of this Journal, the delinquents themselves would be ashamed to find how many physicians can take a Medical Journal, without remembering that it requires money to enable the publishers to comply with their engagements. The indebtedness of each subscriber is a mere song to him, but to us, who have to shoulder the aggregate, the burden is rather more than we feel willing to bear. The editors of this work endeavor to keep its readers advised on every important improvement, or suggested improvement in all the departments of practical medicine, and the works of all civilized countries are laid under contribution for that object. An inspection of the Index, for the numbers for the past six months, which index is contained in this number. will serve to show how large an amount of practical matter is given in a volume of this work. The variety also is very great.

The publishers endeavor to do their duty fully, and we hope that every subscriber who knows of his failure to pay his dues to this Journal, will promptly remit what he owes. The mere name of a subscriber on our books goes but a small way, towards supporting this periodical, and we need "the sinews of men."

### RECEIPTS OF MEDICAL JOURNAL FOR NOV. 1851.

| | |
|---|---|
| Dr. Sparks. York, Ill. | $5 00 |
| J. W. Edes, Parkville, Mo. | 25 00 |
| Shumard, Fort Smith, Ark. | 10 00 |
| C. C. Allen, Chillicothe, Ohio | 5 00 |
| —— Carle, Stillwater Lake, Wis. | 10 00 |
| A. G. Henry, Springfield, Ill. | 5 00 |
| M. Howarth, Soap-Stone, N. C. | 5 00 |
| D. Price, Dresden, Ohio | 15 00 |
| A. B. Palmer, Harrisonville, Mo., | 10 00 |
| R. J. Thompson, Versailles, Ky. | 5 00 |
| J. A. Walton, Clarksvllle, Ark. | 5 00 |
| W. Long, New Maysville, Ind. | 5 00 |
| —— Becker, Metropolis, Ill. | 5 00 |
| J. A. Stewart, Gentryville, Ind. | 10 00 |
| J. C. Depaeu, Pleasant Hill, Ky. | 5 00 |
| —— Johnson, Fredonia, Ky. | 5 00 |

